# The role of lymphatic vessels in corneal fluid homeostasis and wound healing

**DOI:** 10.1186/s12348-023-00381-y

**Published:** 2024-01-22

**Authors:** Karina Hadrian, Claus Cursiefen

**Affiliations:** 1 Department of Ophthalmology, Faculty of Medicine and University Hospital Cologne, University of Cologne, University of Cologne, Cologne, Germany; 2 Center for Molecular Medicine (CMMC), University of Cologne, Cologne, Germany; 3 Cologne Excellence Cluster on Cellular Stress Responses in Aging-Associated Diseases (CECAD), University of Cologne, Cologne, Germany

**Keywords:** Cornea, Lymphangiogenesis, Immune cells, Corneal edema

## Abstract

The cornea, essential for vision, is normally avascular, transparent, and immune-privileged. However, injuries or infections can break this privilege, allowing blood and lymphatic vessels to invade, potentially impairing vision and causing immune responses. This review explores the complex role of corneal lymphangiogenesis in health and diseases. Traditionally, the cornea was considered devoid of lymphatic vessels, a phenomenon known as "corneal (lymph)angiogenic privilege." Recent advances in molecular markers have enabled the discovery of lymphatic vessels in the cornea under certain conditions. Several molecules contribute to preserving both immune and lymphangiogenic privileges. Lymphangiogenesis, primarily driven by VEGF family members, can occur directly or indirectly through macrophage recruitment. Corneal injuries and diseases disrupt these privileges, reducing graft survival rates following transplantation. However, modulation of lymphangiogenesis offers potential interventions to promote graft survival and expedite corneal edema resolution.

This review underscores the intricate interplay between lymphatic vessels, immune privilege, and corneal pathologies, highlighting innovative therapeutic possibilities. Future investigations should explore the modulation of lymphangiogenesis to enhance corneal health and transparency, as well as corneal graft survival, and this benefits patients with various corneal conditions.

## Background

The cornea is the outer barrier of the eye and is, under healthy conditions, transparent and belongs to the few immune-privileged tissues of the organism, meaning it is avascular in the healthy state. If it comes to an injury or infection in the cornea, the corneal avascularity is abrogated if a certain threshold of inflammation is reached [1]. Blood and lymphatic vessels in various constellations (together, isolated, or sequentially) sprout from the vascularized conjunctiva into the cornea which can lead to reduced vision as well as an undesired immune response by invading immune cells. However, previous studies also reported on the beneficial roles of especially lymphatic vessels in the cornea under certain disease conditions [2–4].

This review will focus on the role of lymphatic vessels during corneal wound healing after injuries and infections in regulating corneal fluid balance and will highlight also their beneficial role. The role of lymphatic vessels in the whole ocular compartment was recently reviewed in detail elsewhere [5].

Over the past few decades, a remarkable shift in our understanding of ocular vascular anatomy has occurred, changing long-held beliefs about the complete absence of lymphatic vessels within the eye. The cornea was traditionally considered to be devoid of lymphatic vessels under normal conditions, too, a phenomenon referred to as the "corneal lymphangiogenic privilege" [6]. However, when the cornea experiences trauma or inflammation, both lymphatic and blood vessels can infiltrate the cornea extensively, leading to severe vision impairment and, in some cases, necessitating corneal transplantation, resulting in millions of people suffering from corneal blindness due to a shortage of donor corneas [7, 8].

Furthermore, the unique, densely structured stromal anatomy responsible for maintaining constant dehydration within the cornea can become disrupted by trauma or diseases, potentially allowing vessels to infiltrate and causing corneal swelling, known as edema. In industrialized countries, dysfunction of corneal endothelial cells, which leads to edema and consecutive loss of transparency, is one of the main indications for corneal transplantation [9]. Thus, a nonsurgical approach to reduce corneal edema would be of great therapeutic value to treat corneal blindness worldwide [7, 8].

It is worth noting that early studies demonstrated that corneal edema can occur independently of vascularization [10]. The challenge in detecting lymphatic vessels in the cornea lies in their invisibility during slit lamp examinations [1]. Importantly, their presence doesn't typically lead to visible impairments. As a result, the recognition of lymphatic vessels within the cornea occurred relatively late [6]. Additionally, the absence of reliable markers for identifying lymphatic vessels posed a significant hurdle to research in this area [11]. However, the past 25 years have witnessed significant progress in this field, driven by the discovery of molecular markers such as lymphatic vessel endothelial hyaluronic acid receptor 1 (LYVE-1) [12], prospero homeobox-1 (PROX-1) [13], podoplanin [14], and vascular endothelial growth factor (VEGF) receptor-3 (VEGFR-3) [15]. These discoveries have significantly advanced research on lymphatic vessels within the cornea in the past 25 years [5, 16].

In addition to its unique status as a tissue normally resistant to both lymphangiogenesis and angiogenesis, the cornea is recognized for its immune-privileged characteristics [17]. One example of this immune privilege is the phenomenon known as Anterior Chamber-Associated Immune Deviation (ACAID). ACAID relies, in part, on a specialized subset of macrophages originating from the eye, which induce suppression through Natural Killer cells [18, 19]. This mechanism effectively suppresses antigen-specific, delayed-type hypersensitivity reactions [20]. Notably, research has demonstrated that the immune privilege of the cornea contributes significantly to its resistance to both lymphangiogenesis and angiogenesis [21]. Intriguingly, several molecules play roles in preserving the corneal' angiogenic and immune privileges simultaneously, such as thrombospondin-1 [22] and vasoactive intestinal polypeptide [21].

**Fig. 1:**
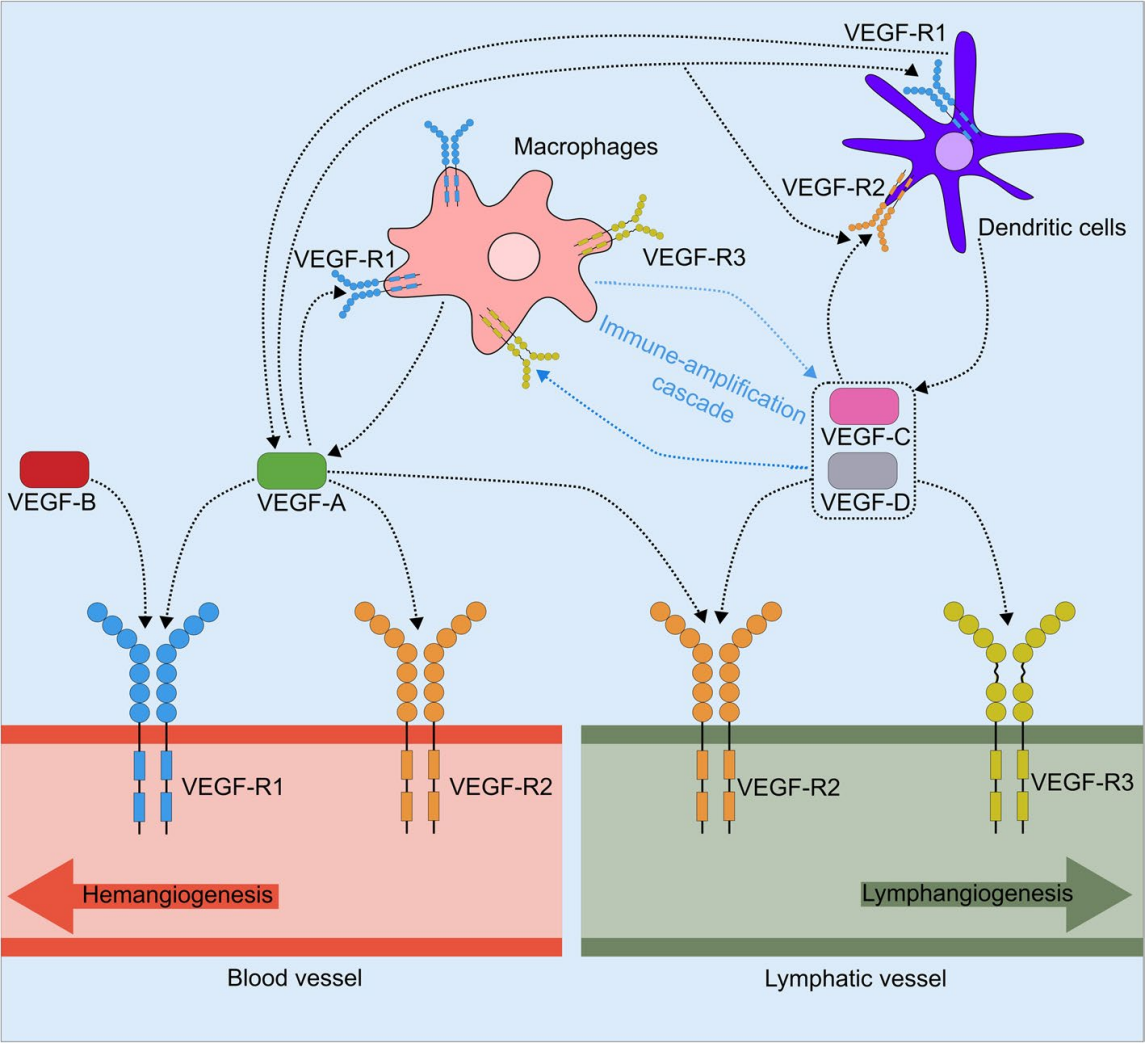
Role of the VEGF family and their receptors on corneal hem- and lymphangiogenesis. Expression patterns of Vascular Endothelial Growth Factor (VEGF) receptors and the binding specificity of their VEGF-ligands (VEGF-A, VEGF-B, VEGF-C, and VEGF-D) are integral to understanding their roles. VEGF-R1 is expressed on both blood vessel endothelium and immune cells such as macrophages and dendritic cells. VEGF-R2 is predominantly expressed on blood vessel endothelium, lymphatic endothelium, and dendritic cells. VEGFR-3 is primarily found on lymphatic endothelium and macrophages. The interactions between VEGF-A, VEGF-B, VEGF-C, and VEGF-D are indicated by dashed arrows. Macrophages play a dual role by not only secreting VEGFs to stimulate angiogenesis but also expressing VEGF-R1 and VEGF-R3. This dual expression facilitates myeloid cell chemotaxis, initiating an "immune-amplification cascade”

Lymphangiogenesis is primarily induced by members of the vascular endothelial growth factor (VEGF) family, notably VEGF-C and -D, which bind to their corresponding receptors VEGF-R2 and -3 [23, 24]. Additionally, the pro-angiogenic factor VEGF-A has also been found to possess the capability to induce lymphangiogenesis [25]. This process can occur in two main ways: indirectly, through the recruitment of VEGF-R1-positive macrophages, which subsequently secrete VEGF-C and VEGF-D, or directly, by stimulating the proliferation of lymphatic endothelial cells [23, 25, 26] (Fig. 1). Additionally, several other growth factors have been identified as contributors to the induction of lymphangiogenesis, including platelet-derived growth factor (PDGF), fibroblast growth factor-2 (FGF-2), hepatocyte growth factor (HGF), and angiopoietin (Ang) [23].

These findings highlight the intricate mechanisms and various factors involved in the process of lymphangiogenesis.

### Breakdown of the immune- and (lymph)angiogenic privilege

In cases of corneal trauma, inflammation, or various diseases, corneal transplantation is often the only option to restore vision. When a corneal allograft is introduced into an avascular host bed, the outcomes are notably promising, with survival rates of approximately 90% after one year and approximately 55% after fifteen years [27]. However, when severe inflammatory stimuli or trauma disrupt the (lymph)angiogenic and immune privilege, leading to neovascularization before transplantation, the one-year survival rate drops to 50% or even lower [27].

Maintaining corneal avascularity is of outmost importance for preserving corneal transparency, which forms the foundation for good visual acuity. However, a range of diseases and surgical procedures can disrupt the cornea's natural privilege against blood and lymphatic vessel growth, resulting in pathological neovascularization of the cornea.

Conditions associated with corneal neovascularization encompass inflammatory disorders, graft rejection following corneal transplantation, infectious keratitis, hypoxia due to contact lens wear, alkali burns, stromal ulceration, and limbal stem cell deficiency. In these scenarios, the delicate balance between pro-angiogenic and anti-angiogenic factors is disrupted, leading to an upregulation of pro-angiogenic factors and a downregulation of anti-angiogenic factors, ultimately resulting in neovascularization [28–30].

When blood vessels grow into the optical zone or cause secondary effects like hemorrhage and lipid exudation through immature and leaky capillaries, they directly compromise corneal transparency. Conversely, lymphatic vessels, which are clinically invisible, do not impact corneal transparency. However, they play a role in various inflammatory ocular surface conditions, including corneal transplant rejection, dry eye disease (DED), and ocular allergy [16, 31]. In these diseases, corneal lymphatic vessels facilitate the migration of antigen-presenting cells (APC) from the ocular surface to regional lymph nodes, triggering undesired immune responses [31–34]. In the healthy cornea, the majority of CD45^+^ cells are macrophages, with dendritic cells constituting only a small fraction [35]. Both macrophages and dendritic cells play a crucial role in the formation of lymphatic vessels during angiogenesis.

### Macrophages and dendritic cells contribute to lymphangiogenesis

Macrophages can promote angiogenesis mainly in three ways. First, the cells secrete VEGF to promote angiogenesis, and also express VEGF-R1 and VEGF-R3, to mediate myeloid cell chemotaxis leading to an “immune-amplification cascade” [25] (Fig. 1). Second, macrophages can express the aforementioned lymphatic markers and thereby become part of the vessel wall and transform into lymphatic endothelium during inflammation [36]. Third, macrophages can act as bridging cells on the tips of sprouting lymphatic vessels, guiding the vessel sprouts to find other vessels for anastomosis [37, 38].

Experimentally it was shown, that the depletion of macrophages with clodronate liposomes reduces the number of lymphatic vessels in mice [39]. These results show that macrophages play an important role in developmental and inflammation-induced lymphangiogenesis.

We also demonstrated, that lymphangiogenesis following a perforating corneal injury, which leads to selective ingrowth of lymphatic vessels [40], relies on the presence of macrophages [41]. Depletion of macrophages significantly reduced corneal lymphangiogenesis, underscoring the crucial role of macrophages in regulating corneal edema and maintaining transparency [41]. The role of macrophages in corneal vascularization is reviewed in detail in [42].

The major role of dendritic cells is the processing and presentation of antigens to activate naive T-cells in the lymph nodes. For that, the dendritic cells mature, enter lymphatic vessels, and travel to the respective lymph nodes. When reaching the wall of the lymphatic vessel, the dendritic cells come in contact with the lymphatic endothelial cell. The lymphatic endothelial cells form oakleaf-shaped overlaps which result in open “flaps” of cells. It is most likely, that the dendritic cells migrate into the vessels via these “flaps” and continue their journey into the draining lymph nodes [43]. Also, dendritic cells interact with lymphatic endothelial cells, which is alleviated by LYVE-1. They further contribute to lymphangiogenesis mainly via the secretion of VEFG-A [44] and VEGF-C [45] and expression of VEGF-receptors (Fig. 1). The frequency of dendritic cells in the cornea massively increases during inflammatory conditions (from 0.4% to 1.1% of all corneal cells) [46].

### Fluid homeostasis of the cornea

The cornea maintains its fluid integrity through two key mechanisms: a barrier function primarily regulated by tight junction proteins like zonula occludens-1 (ZO-1) and a pump-leakage function, which is orchestrated by the active Na^+^/K^+^-ATPase pump mechanism [47]. The Na^+^/K^+^-ATPase pump plays a pivotal role in transporting water and ions from the corneal stroma into the aqueous humor, thereby upholding the corneal thickness and transparency [48]. Dysfunction of the corneal endothelium can result in corneal edema, which, in its advanced stage, may lead to bullous keratopathy [49]. The primary cause of chronic corneal endothelial dysfunction is commonly attributed to Fuchs endothelial corneal dystrophy (FECD) [9].

In contrast to many other organs, such as the skin where lymphatic vessels are well-known for regulating tissue pressure, facilitating fluid drainage, and preventing edema formation [50], the role of corneal lymphatic function in edema resolution remains relatively underexplored, despite confirmation of lymphatics' involvement in various pathologies. As discussed above, the ingress of lymphatic vessels is mostly considered to have a negative influence on the cornea, especially on transplant survival.

### Modulation of lymphangiogenesis can promote graft survival

As described above, VEGFs and their receptors are essential for lymphangiogenesis. Therefore, it stands to reason to interact with the growth factor or its receptors to modulate both hem- and lymphangiogenesis. The role of endogenous regulators of hem- and lymphangiogenesis is discussed in detail here [23]. However, in recent years, the search for new endogenous modulators has continued. For example, the inhibition of cystathionine β-synthase (Cbs) in lymphatic endothelial cells in vitro has been found to reduce proliferation and migration and decrease expression of VEGF-R2 and VEGF-R3, but not VEGF-C and VEGF-D [51]. Also in vivo*,* inflammation-induced lymphangiogenesis is significantly reduced in mice after pharmacological inhibition of Cbs [51].

Angiopoietin-1 (Ang1) for instance, is a growth factor that has the function of generating stable and functional vascularization through the Tie2 and Tie1 receptors. In skin, delayed wound healing is a serious complication [52], therefore, it was shown, that mice treated with cartilage oligomeric matrix protein (COMP)-Ang1 in skin wound healing experiments, showed and accelerated wound closure via stimulation of (lymph)angiogenesis [53]. Vice versa, the blocking of Ang1 might inhibit (lymph)angiogenesis and may delay wound healing in skin, although it promotes graft survival in cornea.

Besides interacting directly with the lymphatic vessels, it is also possible to modify lymphangiogenesis indirectly.

We have recently highlighted the significant role of Interleukin-10 (IL-10)-activated macrophages in the context of inflammatory corneal neovascularization. Specifically, our research has revealed that IL-10, a versatile cytokine with both anti-inflammatory and immune-regulatory properties, governs corneal lymphangiogenesis and the resolution of corneal inflammation through its interaction with macrophages [2]. In mice lacking IL-10, corneal injury led to reduced levels of VEGF-C and diminished corneal lymphangiogenesis. Strikingly, IL-10 deficiency did not impact corneal hemangiogenesis.

Further, the deletion of the Signal transducer and activator of transcription 3 (Stat3) in myeloid cells resulted in reduced corneal lymphangiogenesis and persistent corneal inflammation following injury. However, the influence of IL-10 on graft survival is not clear yet [54–56]

### Corneal trauma and inflammation are often followed by lymphangiogenesis and corneal edema

Numerous types of corneal injuries are linked to the development of corneal edema, yet the involvement of lymphatic vessels has only been extensively studied in a limited subset of these injuries. Recently, it was observed, that full-thickness penetrating corneal incisions lead to a selective growth of lymphatic vessels into the cornea with an acute corneal edema and increased opacity [3, 40]. Additionally, we could demonstrate, that blocking lymphangiogenesis with an anti-VEGF-R3 antibody leads to delayed corneal drainage and tends to prolong corneal opacification [3]. These findings suggest that lymphangiogenesis is relevant, particularly in resolving acute corneal edema, therefore lymphangiogenesis can have a positive effect, which was also shown by Narimatsu and colleagues [4]. In bacterial keratitis, especially in its late stage, depletion of macrophages has been shown to reduce lymphangiogenesis and delay the resolution of corneal edema [4], also indicating, that corneal lymphangiogenesis may offer potential benefits for resolving corneal edema in such cases.

Interestingly, in corneal herpes simplex virus (HSV)-1 manifestations, which also leads to corneal edema, lymphangiogenesis is induced rather by VEGF-A secreted by infected epithelial cells than by macrophages [57].

### Modulation of lymphangiogenesis to promote a faster edema resolution

The interconnection between lymphatics and edema in corneal pathologies is widely recognized, and further exploration of this relationship should be a key focus in future research. It remains to be demonstrated whether enhancing lymphangiogenesis, leading to increased lymphatic vessel growth, could expedite the resolution of corneal edema. If certain factors can selectively stimulate lymphangiogenesis without affecting hemangiogenesis, it might be possible to enhance these factors' availability, potentially increasing the resorption of corneal edema. As described above, mice tread with COMP-Ang1 showed improved wound healing in skin [53]. A similar usage in corneal is conceivably to improve corneal edema resolution.

However, research on the transcription factor Nuclear Factor of Activated T Cells 5 (NFAT5), also known as Tonicity-Responsive Enhancer Binding Protein (TonEBP), which induces the transcription of VEGF-C, has revealed intriguing findings. Knocking down NFAT5 in mice resulted in a faster resolution of corneal edema following perforating corneal injury [58]. Notably, lymphangiogenesis after such injuries remained unaffected. Yet, in vitro studies involving bone marrow-derived macrophages from NFAT5 knockdown mice demonstrated an increased rate of pinocytosis in inflammatory macrophages, potentially contributing to a more rapid edema resolution after perforating corneal injury [58]. These findings suggest that not only can lymphangiogenesis itself be a target for intervention, but also immune cells.

## Summary and conclussion

**Fig. 2:**
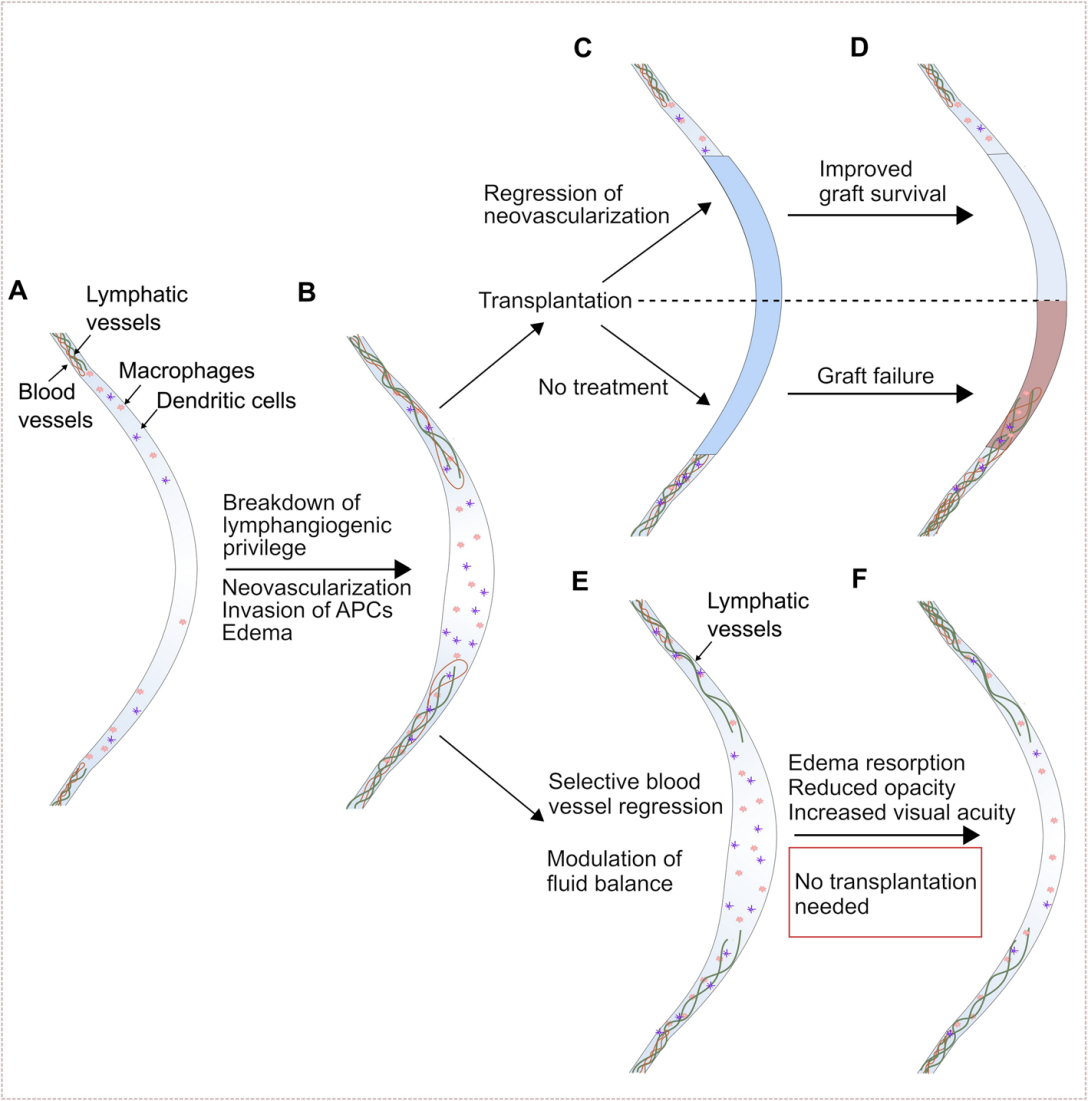
The possible role of lymphatic vessels in corneal fluid homeostasis and wound healing to avoid corneal transplantation. The healthy cornea **A** is avascular and contains only a few immature antigen-presenting cells (APCs). If it comes to a breakdown of the lymphangiogenic privilege **B** neovascularization occurs, accompanied by an invasion of APCs and possibly edema, often leading to the need for transplantation **C** If the graft is transplanted into a pre-vascularized and inflamed bed, it often leads to graft failure. However, graft survival can be improved by a regression of neovascularization before transplantation. Possibly, a selective regression of blood vessels but not lymphatic vessels and an additional (molecular) modulation of fluid balance **E** can lead to a fast edema resorption, which reduces opacity and restores visual acuity **F** As a consequence, no transplantation is needed

The cornea's unique attributes, including avascularity, transparency, and immune privilege, play crucial roles in maintaining vision and ocular health (Fig. 2A). However, when these privileges are compromised due to injuries or diseases, it can result in pathological processes, such as corneal neovascularization and edema (Fig. 2B).

Recent advancements in understanding the presence of lymphatic vessels within the cornea have expanded our knowledge of its intricate biology. While these lymphatic vessels were initially thought to be absent, molecular markers have revealed their existence under certain conditions, their role in both corneal pathology and potential therapeutic interventions has been explored.

Corneal neovascularization is of significant concern, particularly in transplant scenarios where graft survival rates are compromised when neovascularization occurs before transplantation (Fig. 2C). However, graft survival can be increased by a regression of neovascularization before transplantation (Fig. 2D). Furthermore, macrophages and dendritic cells play pivotal roles in the angiogenic and lymphangiogenic processes, further emphasizing their importance in maintaining corneal transparency.

Corneal edema, another complication arising from corneal injuries and diseases, has been associated with lymphatic vessel growth. While traditionally seen as a negative, recent research suggests that selective corneal lymphangiogenesis might expedite edema resolution without compromising transparency and thus be beneficial for corneal health (Fig. 2E/F).

Understanding these complex interactions and the potential to modulate lymphangiogenesis opens up future research and therapeutic interventions. Targeting immune cells or specific factors that influence lymphatic vessel growth may offer innovative strategies to enhance graft survival and expedite edema resolution.

In conclusion, the cornea's immune privilege, avascularity, and (secondary) lymphatic vessels are essential components of ocular health (and disease). Continued exploration of their relationships and potential interventions hold promise for improving outcomes for patients with various corneal conditions, ultimately preserving and restore vision.

## Data Availability

N/A.

## Abbreviations

ACAID: Anterior Chamber-Associated Immune Deviation
Ang: Angiopoietin
APC: Antigen-presenting cells
COMP: Cartilage oligomeric matrix protein
Cbs: Cystathionine β-synthase
DED: Dry eye disease
FECD: Fuchs endothelial corneal dystrophy
FGF: Fibroblast growth factor
HGF: Hepatocyte growth factor
HSV: Herpes simplex virus
IL: Interleukin
LYVE-1: Lymphatic vessel endothelial hyaluronic acid receptor 1
NFAT5: Nuclear Factor of Activated T Cells 5
PDGF: Platelet-derived growth factor
PROX-1: Prospero Homeobox-1
STAT: Signal transducer and activator of transcription
TonEBP: Tonicity-Responsive Enhancer Binding Protein
VEGF: Vascular endothelial growth factor
VEGF-R: Vascular endothelial growth factor receptor
ZO-1: Zonula occludens-1
